# Bibliographical Department

**Published:** 1881-06

**Authors:** 


### BIBLIOGRAPHICAL DEPARTMENT.

"Nothing extenuate, nor set down aught in malice."

*A System of Oral Surgery: Being a Treatise on the Diseases and Surgery of the Mouth, Jaws, and Associate Parts. By James E. Garretson, M. D., D. D. S., Dean of the Philadelphia Dental College; Surgeon in charge of the Hospital of Oral Surgery; Member of the Philadelphia County Medical Society; Member of the Philadelphia Pathological Society; Honorary Member of the Delaware County Medical Society; Honorary Member of many Dental Societies; late Oral Surgeon to the University of Pennsylvania; late Lecturer on Anatomy and Surgery in the Philadelphia School of Anatomy, etc., etc. Illustrated with numerous steel plates and wood cuts. Third edition. Thoroughly revised, with additions. Philadelphia: J. B. Lippincott & Co.; London:* 16 *South Hampton street, Strand,* 1881.

In this splendidly gotten-up volume of 916 pages we have all that is really worth knowing upon the subject of oral surgery. There are very few men who write with the same degree of ease and clearness as does Dr. Garretson, or who have had better opportunities and practical experience in the specialty to which he has devoted thirty years' of active practice. The text is full and explicit, and the cuts and engravings render everything clear and easy of comprehension on the practical subjects to which they relate. The type and binding reflect credit upon the enterprising publishing house from which the volume emanates. It is a book that should be in the hands of all surgeons as well as dentists.

